# Adherence to Oral Anticancer Medications: Evolving Interprofessional Roles and Pharmacist Workforce Considerations

**DOI:** 10.3390/pharmacy6010023

**Published:** 2018-03-08

**Authors:** Gennaro A. Paolella, Andrew D. Boyd, Scott M. Wirth, Sandra Cuellar, Neeta K. Venepalli, Stephanie Y. Crawford

**Affiliations:** 1 Naval Medical Center San Diego, United States Navy, San Diego, CA 92101, USA; gpaolella@comcast.net; 2 Department of Biomedical and Health Information Sciences, University of Illinois at Chicago, Chicago, IL 60612, USA; boyda@uic.edu; 3 Department of Pharmacy Practice, Ambulatory Pharmacy Services, University of Illinois at Chicago, Chicago, IL 60612, USA; swirth1@uic.edu (S.M.W.); scuell1@uic.edu (S.C.); 4 Department of Medicine, Hematology/Oncology, University of Illinois at Chicago, Chicago, IL 60612, USA; nkv@uic.edu; 5 Department of Pharmacy Systems, Outcomes and Policy, University of Illinois at Chicago, Chicago, IL 60612, USA

**Keywords:** communication, team effectiveness, medication adherence, oncology workforce, oncology pharmacist, applied theory, systems science

## Abstract

Interprofessional care is exhibited in outpatient oncology practices where practitioners from a myriad of specialties (e.g., oncology, nursing, pharmacy, health informatics and others) work collectively with patients to enhance therapeutic outcomes and minimize adverse effects. Historically, most ambulatory-based anticancer medication therapies have been administrated in infusion clinics or physician offices. Oral anticancer medications (OAMs) have become increasingly prevalent and preferred by patients for use in residential or other non-clinic settings. Self-administration of OAMs represents a significant shift in the management of cancer care and role responsibilities for patients and clinicians. While patients have a greater sense of empowerment and convenience when taking OAMs, adherence is a greater challenge than with intravenous therapies. This paper proposes use of a qualitative systems evaluation, based on theoretical frameworks for interdisciplinary team collaboration and systems science, to examine the social interactionism involved with the use of intravenous anticancer treatments and OAMs (as treatment technologies) by describing patient, organizational, and social systems considerations in communication, care, control, and context (i.e., Kaplan’s 4Cs). This conceptualization can help the healthcare system prepare for substantial workforce changes in cancer management, including increased utilization of oncology pharmacists.

## 1. Introduction

Pharmacologic anticancer therapies are increasingly shifting from clinic-based infusions to orally-administered medications by patients and/or their informal caregivers, thereby changing patient and health professional roles, responsibilities, and priorities [1,2]. Awareness of the evolving interprofessional roles, as well as social and technological interplay, can facilitate improved adherence with prescribed anticancer therapeutic regimens. This paper describes a conceptualization of the social interactionism dynamics involved in cancer care management with emphasis on the changing workforce requirements for pharmacists.

An estimated 15.5 million Americans are living with cancer, and 1.69 million Americans were expected to be diagnosed with some form of cancer in 2017 [3,4]. The historical mainstay of pharmacologic treatment has been ambulatory, infusion-based chemotherapy. While oral formulations (e.g., tablets and capsules) of anticancer drugs have been on the market for decades, Food and Drug Administration (FDA) approval of capecitabine in April 1998 marked the start of a new wave of major advancements in oral anticancer therapies in the U.S., including a flurry of approvals of targeted oral anticancer medications (OAMs) that continues to this day. Tyrosine kinase inhibitors, histone deacetylase inhibitors, vascular endothelial growth factor (VEGF) inhibitors, and mammalian target of rapamycin (mTOR) inhibitors [5,6,7] are just a few examples of novel OAMs. One-third of anticancer therapies on the U.S. market are available in oral formulations [8,9], and OAMs represent 25–30% of anticancer drugs in pipeline development [7]. When clinically appropriate and equally efficacious [10,11], patients prefer OAMs over intravenous (IV) therapy because OAMs provide patients with a greater sense of control over their care, convenience, improved quality of life, decreased lost time traveling to clinical appointments, reduced need for invasive procedures, and fewer distractions from work and social activities [1,12,13,14,15]. Safe handling of all anticancer medications, including OAMs, is important for patients and their caregivers, in addition to healthcare professionals [16,17,18,19,20].

Medication adherence is the degree to which patient behavior and action corresponds with the agreed upon therapeutic regimen from the healthcare practitioner with respect to timing, dosage, and frequency [21,22]. Consequences of nonadherence are well documented in chronic diseases and lead to increased healthcare utilization, e.g., unnecessary doctor visits, emergency room visits, and hospitalizations [23,24,25], resulting in additional costs of $100 to $300 billion annually within the U.S. [26]. Medication adherence is an indicator of therapeutic response in cancer care management [27]. For instance, one study found that patients with chronic myeloid leukemia with >90% adherence to imatinib demonstrated increased probability of a six-year 3-log reduction of disease compared to patients with adherence levels ≤90% (94.5% vs. 28.4%, *p* < 0.001) [28]. Consequences of non-adherent behavior also include development of resistance, increased burden for patients and caregivers, disease progression, and death [10].

While patients with cancer display higher motivation towards medication adherence, challenges remain [23,24,27,29,30]. Adherence estimates for patients taking OAMs range from less than 20% to 100%, but are typically between 50% and 90% [7,30,31,32,33]. OAM adherence levels are lower than IV anticancer therapies [14]. Reasons for nonadherence to OAMs include complex treatment regimens, intolerable side effects, inconvenient clinic visits, unfilled prescription refills, inadequate supervision, problematic patient–clinician communications, socioeconomic factors, and high medication costs [1,7,12,27,34,35]. Additionally, an estimated 10% of patients on newly-prescribed OAMs never pick up the prescription to start treatment, which could lead to dire consequences [36,37].

## 2. The Oncology Workforce

The swift rate of OAM development, FDA approval, and prescribing over the past 15 years has changed ambulatory-care service modes. Healthcare professionals should anticipate operational system changes and needed strategies and procedures (e.g., communications, task flow, relationship building and team coordination) to effectuate improved adherence, planned therapeutic outcomes, and high-value care [17,38,39,40,41,42]. Increased oncology services and care provided by nurses, nurse practitioners and physician assistants have been highlighted [41,43]. Sessions and colleagues noted increased utilization of highly knowledgeable, specially-trained oncology pharmacists can help optimize cancer care and help address projected workforce needs in cancer management [44]. Those authors noted the extensive knowledge that oncology clinical pharmacists bring to the healthcare team, such as expertise in pharmacotherapy, toxicities, monitoring, and pharmacoeconomics. Through contributions to multidisciplinary teams, clinical oncology pharmacists should be prepared for the changing healthcare landscape and new practice models to facilitate improved outcomes for patients with cancer [45].

The scope of practice document from the Hematology/Oncology Pharmacy Association noted the integral role of hematology/oncology pharmacists in cancer care teams in a variety of roles [46,47]. Common services and care provided by oncology pharmacists include developing therapeutic plans, collaboration with oncologists in medication selection/adjustment, adverse drug reaction prevention and monitoring, laboratory test ordering and monitoring, drug compounding, dispensing, supply management, patient counseling, patient/family and provider education, addressing drug insurance requests, research, and assisting in the development of evidence-based guidelines [48,49,50,51,52].

Currently, most pharmacists have limited formal education and training in hematology and oncology. In the U.S., student pharmacists typically receive a block of lectures on oncology medications in the required core course on therapeutics or a standalone required course, and typically only about 30% are exposed to oncology practice through four-to-six week Advanced Pharmacy Practice Experience clerkship electives [53]. The pathway to becoming an oncology pharmacist often includes on-the-job training. Knowledge and skills can be obtained through specialized postgraduate pharmacy residency training or other structured traineeship, and a competency-based approach has been called for in efforts to expand the workforce in cancer care [54].

The highest level of credentialing for pharmacists in cancer care is recognition as a Board of Pharmacy Specialties (BPS) board certified oncology pharmacist (BCOP), a designation that requires extensive postgraduate training in oncology pharmacy and passing of the Oncology Pharmacy Specialty Certification Examination (and periodic recertification requirements) [55]. A total of 2265 pharmacists were designated as BCOP in 2016 [56], which represented less than one percent of the approximately 305,510 pharmacists in the U.S. [57]. Some states recognize pharmacists as providers that are able to prescribe or administer drugs under a scope of practice or collaborative practice agreement. With relatively few pharmacists recognized as oncology specialists and the huge demand for oncology pharmacist services, workforce solutions are needed to achieve high-value patient care. Subsequent sections of this article describe collaborative models (with the patient, within the profession, and via interdisciplinary collaborations) that are needed for optimal patient care management.

## 3. Patients as Cancer-Care Providers

A cautious approach to cancer therapy management is warranted. Patients taking OAMs (in their home or other non-institutional or non-clinic settings) are consuming drugs with a high potential for toxicity [58]. OAMs can cause intolerable side effects and/or possible adverse effects that necessitate ongoing monitoring and assessment [1,59]. OAMs require special handling and can involve complicated dosage regimens. For example, some patients receive OAMs where two medications are to be taken on given days, but only one medication on other days, or with on–off cycling in dosage regimens (mono or combination therapy). Accurate dosing may necessitate numerous pill combinations. Some OAMs must be taken with food, while others should not be taken within several hours of eating. Patients with cancer are often prescribed additional medications to treat multiple comorbidities such as hypertension, hyperlipidemia, depression, and pain, or shorter-term drug regimens to manage side effects of anticancer medications such as chemotherapy-induced nausea and vomiting. The totality of dose, schedule, and administration of medications becomes extremely complicated, even in a healthcare proficient patient. Cancer patients with low health literacy may experience greater written and spoken communication difficulties, be at higher risk for self-medication errors, and benefit from counseling and education on OAM use from healthcare professionals who are sensitive to their communication needs [60,61].

Preventable errors with the potential for patient injury with home-based cancer medication management have been observed at rates similar to or higher than what occurs in hospitals [62,63]. Patients with chronic diseases are required to be “effective self-managers of their illness” [64] and co-creators in care management [65]. Patients must be well-managed by clinicians for early detection of adverse effects that merit counseling and/or dosage or medication adjustments or other untoward conditions that raise the likelihood of non-adherence and poor patient outcomes [66]. Patient education and behavioral interventions can be vital in efforts to achieve treatment goals with OAM regimens, and interdisciplinary care efforts should be well planned and evaluated to promote medication adherence, which should improve cancer survival. A conceptualized model of the interactive patient and healthcare professional roles across the course of treatment can foster improved patient-centered care [40]. Oncology pharmacists are essential for expedition of initial therapy start, subsequent therapeutic management decisions, foreseeable prevention of adverse effects, and ongoing provision of high quality, patient centered and evidence based care [44,47,67,68]. Integration of oncology pharmacists into the cancer care team and healthcare model is vital for effective delivery of care for patients [69]. Demonstrated examples include oncology pharmacists’ impact with respect to patient care services, supportive care services, and interventions on inappropriate polypharmacy and potentially inappropriate medication use [20,70,71,72,73].

## 4. Cancer Care Management Using a Theoretical Lens

D’Amour and colleagues reviewed published conceptual frameworks for interdisciplinary team collaboration, identifying four major commonalities—“sharing” (shared responsibilities, decision-making, data, interdisciplinary perspectives), “partnering” (based on effective communication and trust), “interdependency”, and “empowerment” of team members regardless of title or function [74]. While these considerations underpin the essence of interprofessional collaboration, a proactive practical model is needed to ensure smooth care processes and increase adherence for patients taking OAMs to maximize therapeutic effectiveness and minimize safety concerns and delayed drug therapy from nonadherence [12,66]. Adaptive systems theory for dealing with uncertainties in the system [75] and systems-based practice (knowing how an individual action affects functioning and outcomes in other aspects of the system) in a complex healthcare environment or clinical microsystem subsumes core elements of “interdependency” and “interrelationships”—such as interdisciplinary sharing and partnering, as well as individual empowerment [76].

Systems science provides qualitative methods for evaluating healthcare delivery that can be applied in the pharmacological care of oncology patients. “Systems science” refers to the interactive, dynamic, and multilevel (individual, community, and societal) interrelated pathways that should be considered in a macro view of problem solving [77]. Theoretical knowledge from systems science can be contextualized by considering the social systems of people and how they interact with others and with their environments, i.e., “situated cognition” [78]. While the need for IV site access with attendant care by healthcare professionals is obviated with patient self-administration of OAMs [58], the need for coordinated care persists. By comparatively assessing oral and IV treatment modalities using a qualitative systems evaluation, researchers and members of the oncology care team can redesign systems for optimal patient care. A qualitative systems evaluation considers the social dimension, which includes individual factors (including cultural issues) and both institutional/organizational and trans-organizational levels (i.e., beyond the cancer clinic or oncology pharmacy in our application) in the workflow and process flow [79]. Social interactionism weaves the interplay between organizational systems, social interactions, and technology [78,80].

In applying social interactionism, “Kaplan’s 4Cs” (as originally developed by Bonnie Kaplan) denote sociotechnical relationships within a healthcare setting and changes in the ways departments linked by computer systems communicate, the impact on management and organizational control, and care delivery prompted by a technology within the organizational context. In short, Kaplan’s 4Cs assess communication, care, control, and context [81]. “Communication” refers to how systems implementation of the technology alters information flows within a system, including patient-provider communications. Communication is a critical aspect of healthcare and technology as new care delivery techniques interrupt existing communication pathways. The “care” segment conveys how the technology impacts service delivery and health outcomes. Conclusions as to how patients are affected are limited to the care segment. “Control” describes how a technology affects the workflow of users and management. Control is the sense that the provider or patient can make choices or actions that influence the health outcomes. “Context” pertains to the circumstance in which a technology is adopted (i.e., systems implementation in the specific organizational practice setting and how decisions are made with the knowledge of the individuals). Context is the overall situation of the patient, care plan, delivery of care, and healthcare team. Context allows patients to receive personalized treatment instead of just following a flowchart. Application of Kaplan’s 4Cs to dynamic social systems processes and evolving technologies can help provide an evidence-based model in evaluations of systems and outcomes [82]. For example, the social interactionist framework of Kaplan’s 4Cs was used in a small European study to evaluate patient survey responses and general practitioner interviews about usability of a smartphone app to improve adherence for patients with diabetes [83,84].

This paper considers IV anticancer therapies and OAM treatment modalities as ‘technologies’, different from but analogous to the health informatics technology originally described in the context of social interactionism. Applying Kaplan’s 4Cs to an entire treatment modality (i.e., OAMs and IV anticancer therapies) substantially expands the scope of this evaluation tool. Use of Kaplan’s 4Cs represents a practical application of social interactionism, i.e., small theoretical programmatic application for quality improvement [85]. The enhanced roles that patients and caregivers assume with prescribed OAMs, as well as patient experiences, warrant consideration of the different aspects encompassed by the 4Cs. Table 1 summarizes the comparisons described below.

## 5. Communication

Effective communication in oncology care provision is defined as the open expression of opinions, concerns, and questions in the form of dialog among patients and the healthcare team; nonverbal cues also demonstrate levels of message understanding, comfort, and emotion [86]. Patients receiving infusion therapy relay concerns as appropriate, often via a real-time interactive question-and-answer format with their practitioners, as well as a visual assessment of body language. Concerns are typically evaluated through interdisciplinary teamwork by oncology nurses or pharmacists and shared with the appropriate health professional(s) within the same facility or center. Thus, patients with cancer receiving IV therapy have extended contact with healthcare professionals within the same setting on an ongoing basis. This allows patients to communicate face-to-face, receive advice directly from providers during infusion times, and build relationships with providers. In one study, time spent discussing health-related quality of life between oncologists and patients correlated with the number of previous appointments [87]. Regardless of setting, patient-centered interpersonal communication in oncology care is essential in delivering a high quality patient experience [88,89,90]. Clinicians should solicit and provide feedback to ascertain if messages are received as intended. Topics addressed between clinicians and patients (and possibly their family or other caregivers) typically include cancer treatment plan, need for treatment adherence, anticipated side effects, monitoring and reporting adverse events.

In contrast to IV therapy, OAM management offers opportunities and new challenges to build patient-health care provider communications and support systems. For example, patients taking OAMs require extensive educational interventions and counseling focused on drug administration instructions, side effects and symptom management, possible dosage adjustments per advice of the oncologist, storage and safe handling, drug interactions, and interdisciplinary supportive care [16,19,20]. This extensive educational intervention and counseling may be provided by oncology pharmacists, however that is not always the case. In some instances, oncologists or their staff initiate patient-centered education about OAMs [12] that may require additional work resources. Some literature suggests the complexity of communicating the necessary breadth and depth of information about OAMs to patients requires additional time and effort from mid-level providers, often requiring role expansion without organizational compensation for such services [1,12]. Even within the inpatient setting, medical information shared between providers during the handover process has been found to be inadequate (i.e., restricted and incomplete) [91]. Barring effective communication with patients and/or pharmacists, prescribers may be unaware of actual usage dates for OAM regimens. Improved communication and interprofessional collaboration in sharing knowledge about start-up and follow-up dates of OAM regimens (dispensing and actual patient behavior) are needed for effective monitoring of therapeutic effects and safety concerns [12]. Oncologists, nurses, and pharmacists need to be in sync with this information, as with IV therapy, to optimize OAM therapy. In addition, updated clinic or office procedures may be needed to establish quality initiatives in documenting patient start dates and discontinuation dates for OAMs [92]. Electronic tools, such as mobile apps, may help pharmacists and other healthcare professionals in providing patient education and assessing adherence, while assisting patients in medication management [15,93,94]. Published interventions include interactive telephone interventions spearheaded by nurses, medication management appointments combined with side effect monitoring by pharmacists, patient-centered applications to increase OAM adherence, and creation of an entire clinic solely dedicated to OAMs [34,93,95,96]. Despite the potential of such initiatives to enhance patient care, systematic constructs (i.e., communication flow, care delivery, social dynamics, and work environment) that coexist with an intervention may alter or even compromise effectiveness. Applying systems science-based theoretical frameworks can address and lead to solutions that overcome such barriers as patient-centered factors would be considered along with institutional processes and social interaction systems beyond the organization. Fundamentally, the interpersonal communication models between the infused therapies and OAMs have undergone a significant change. The communication with the pharmacist will be the most current information and the last interaction unless the patient initiates additional communication with the care team. If the patient is anxious, has questions or is hesitant, then the oncology pharmacist (or possibly other pharmacist such as those employed in specialty pharmacies) will be the last healthcare practitioner to engage and/or communicate concerns with the larger healthcare team. Specifically, the pharmacist is positioned to identify medication issues before, during, and after initiation of OAM therapy. Issues such as medication adherence, medication changes, and medication tolerability in a cancer patient are dynamic variables that require the careful attention and subsequent communication to the healthcare team.

## 6. Care

Patients prescribed OAMs need to realize the critical role they play in self-care. Tasks historically satisfied by oncologists, nurses, or oncology pharmacists (in physician offices or associated clinics) are relegated to patients and/or caregivers who are distanced from treating clinicians during drug administration. This represents a material shift in the management of cancer care from a provider-driven intermittent infusion model to a chronic disease model [97]. Studies are needed to assess the effects this transition has on cancer patient outcomes and care delivery. At present, trends observed in other chronic diseases help guide therapy management.

Outpatient oncology centers provide patient care services and more safety checks in a structured environment for drug administration, monitoring, and supportive services. Patients receiving IV therapy have generous opportunity to take advantage of these services given the increased frequency and routine time required for infusion appointments. The frequency of care allows clinicians to have multiple patient evaluations for comparison purposes as well as a familiarity with all staff and clinicians of a center. While all essential care is documented, the interaction frequency can lead to more opportunities to better understand the social context of the patient (e.g., their caregivers and social support, transportation issues).

Although patients taking OAMs might receive oncology care in the same facility where the medicines are dispensed, the oral medication self-administration reduces opportunities for direct observation and care provision. Consequently, the roles of patients and/or informal caregivers in drug administration and monitoring is expanded. This could lead to similar diminished treatment outcomes from nonadherence as observed within other chronic diseases [7]. To prepare themselves better for (self-)administration of OAMs, patients and caregivers might seek care from health professionals outside of the practitioners actively involved in their direct treatment in oncologist offices and cancer clinics. For example, patients might direct questions to an extended network of practitioners providing base support in other ways (e.g., spiritual advisors, social workers, friends and family) or seek social support from a wider community network such as patient advocates, friends, or medical information staff in the pharmaceutical industry [40,98,99]. Patients who receive care external to an oncology center may be more likely to report adverse events to their primary care physicians or community pharmacists [100]. Many professionals outside of an oncology practice may have limited or no experience with OAMs [101]. Regardless of experience level, coordinating multidisciplinary cancer care becomes inherently more difficult as the number of providers increases [1].

Patients taking any form of chemotherapy or OAMs are at heightened risk of experiencing side effects or life-threatening adverse effects and should be educated about symptoms and the need to report promptly to their oncologist, oncology nurse, or pharmacist. It is imperative that patients know how to appropriately react, when to seek medical attention, and, if necessary, seek emergency care. This is especially true for patients taking OAMs as they are self-managing drug therapy largely away from oncology care providers. Drug toxicities have greater potential to go untreated until future clinic appointments. Oncology pharmacists are in a unique position to identify OAM medication side effects (or more serious adverse effects) because of repeated access to the patient. Interactions with pharmacists may occur either at the point of OAM dispensing or at the clinical site.

## 7. Control

Intravenous therapies are administered in environments where members of the healthcare team have almost complete control over the therapy administered. Attending clinic appointments as scheduled is largely under patient control if access to services is possible. Healthcare systems typically implement rigorous workflow measures to ensure patient safety and mitigate medication errors [98]. Organizations can ensure that health professionals comply with institutional policies based on insurance reimbursement requirements, care guidelines, and other organizational policies. Medication reconciliations and provider notes can be easily accessed in the patient record for oncologists to make care decisions, and organizations can more readily assess quality assurance. Such organizational control becomes shared with patients and caregivers when an OAM is prescribed.

As a result of the complexity of OAM therapies, accreditation standards for specialty pharmacies have been developed by organizations such as the Accreditation Commission for Health Care (ACHC), Center for Pharmacy Practice Accreditation (CPPA), and URAC [102,103,104]. In general, these accreditation standards improve medication management and improve quality of care provided by pharmacists [105].

Care regimens for patients taking OAMs require better relations and coordination among diverse healthcare practitioners. The oncology pharmacy workforce should be well trained (e.g., certification as BCOP and/or enhanced professional development and continuing education for other pharmacists engaged in oncology care) to enhance this coordination by providing consistent management across all areas of medication provision and monitoring. This would allow for the same level of care when medications are dispensed from outside pharmacies (i.e., specialty pharmacies not located within the same institution), but medication management is coordinated by clinic-based oncology pharmacists. A less fragmented, more seamless transition of care across all levels of OAM management (education, dispensing, professional communication, follow-up) may eliminate confusion for patients, minimize the potential for errors, and increase confidence in their ability to appropriately self-administer their OAMs. Pharmacists have the opportunity to improve this process through enhanced communication with outside providers or pharmacies, the patient, and the primary care team.

Greater control over drug administration with OAMs can instill feelings of empowerment among patients [13]. Essentially, patients must become effective self-managers for their disease. Providers might have limited knowledge of patient medication adherence aside from word of mouth and review of refill histories.

## 8. Context

Interdisciplinary interactions among oncologists, nurses, pharmacists and others within medical centers and/or oncology-clinics are generally well facilitated for patients receiving IV medications. All individuals making decisions regarding the infused medications have enough information to provide adequate context about the patient and the status of the cancer to decide the next step. Health system infrastructures for care coordination provides less context for patients taking OAMs. Patients might need to obtain OAMs through a specialty pharmacy or other pharmacy setting. Different pharmacy outlets have varying services available pertaining to adherence monitoring, medication therapy management, prior authorization experience, and patient assistance programs [12]. Dispensing of OAMs in data-poor environments (e.g., without the ability to access patient medical history, care plan) and in settings where pharmacists are not specifically trained in managing complex OAM therapies represents a challenge to successful patient outcomes, given that providers must be able to provide adequate patient counseling and education in the absence of essential context and patient information.

Additionally, reimbursement for OAMs is managed through different payment schemes than IV therapies. In non-institutional settings, IV anticancer drugs are usually billed through major insurances as part of the medical benefit plan using a fee-for-service system. A highly substantial percentage of revenue in private oncology practices stems from infused therapy administration and monitoring [1]. Conversely, covered OAMs are billable through the prescription drug benefit, usually administered by third-party pharmacy benefits managers (PBMs) [12,106]. Patients taking OAMs have historically incurred increased out-of-pocket cost sharing expenses for deductibles, copayments, and coverage gaps [107]. High costs of OAMs can be prohibitive as typical prescription costs for monthly courses of therapy are $1500 for traditional oral chemotherapy and from $3000 to over $11,000 per month for targeted OAMs [1,108,109,110,111]. As of December 2017, 43 states and the District of Columbia enacted “parity laws” placing caps on out-of-pocket costs for OAMs or requiring health plans to provide more equitable cost-sharing provisions to ensure comparable coverage for both IV and OAM treatments [112,113]. State parity laws, however, have not consistently demonstrated patient protection from high out-of-pocket costs for OAMs [114]. Federal U.S. parity legislation, i.e., H.R. 1409, Cancer Drug Parity Act of 2017 [115], has been proposed to mandate that group and individual health plans offer patient cost sharing options that are at least equivalent for OAMs as for IV therapies under the medical benefit. Economic evaluations are inconsistent with respect to cost-effectiveness of targeted OAMs versus targeted IV therapies of comparable efficacy [12,107,110,114].

PBMs commonly institute administrative requirements (e.g., prior authorization) from the pharmacist or oncologist prior to initiating and continuing OAM coverage. Patients taking OAMs have increased responsibility in partnering with their pharmacists and oncologists to manage timely drug procurement as treatment delays might occur in the payment authorization processes. The procedures needed for timely drug procurement and refills may be confusing to patients without adequate education by healthcare practitioners. The change in contextual awareness needs to be explicitly included in the oncology pharmacist’s plan and collaboration with the oncology team. Non-specialist pharmacists in other ambulatory settings may need additional oncology training and education (in OAM regimens, continuity of care as team members, and handling/disposal of OAM drugs) in order better coordinate specialized clinical care with reimbursement and procurement strategies [116,117].

## 9. Conclusions

Optimal care for patients with cancer involves team science, including consideration of complex social and organizational phenomena in the interdependent, multilevel healthcare systems [118]. Prescribing oral anticancer therapies presents larger implications on care delivery than merely altering the route in which a drug is administered. Healthcare systems will become increasingly incentivized to provide adherence-boosting initiatives and monitoring for patients taking OAMs as value-based reimbursement models take precedence over fee-for-service models [119,120]. Utilization of new technologies in complex healthcare systems is dependent upon good interprofessional team performance and adaptable shared mental models to address potential performance failures [99,121]. The Kaplan 4C evaluation highlights mutual relationships across treatment modalities, people, tasks, and organizational and social structures. The Kaplan 4Cs can be used as a practical application for planning care continuity and appreciating the evolving requirements on both the patients and the healthcare system and creating new paradigms for pharmacists. In collaboration with medical oncologists and other team members, oncology pharmacists should participate in efforts to optimally manage cancer medications and to minimize or prevent medication-related adverse events.

Changes in oncology care will have implications for new directions in the use of workforce and in workforce training. Oncology pharmacists have the ability to play a central role in the management of OAMs due to their extensive knowledge in medication administration, monitoring, and reimbursement. Their impact will be determined by their ability to be proactive in providing patient education, coordinating care among healthcare providers and dispensers, and their communication and follow-up with the patients who are in control of administering OAMs. To help meet patient demand, the oncology pharmacist workforce needs to help educate non-specialist pharmacists and other healthcare professionals. Oncology pharmacists should collaborate and share information with others involved in providing oncology care to patients, such as pharmacists practicing in specialty clinics. In conclusion, oncology pharmacists have an increased role in interdisciplinary patient care, patient care management, educational intervention and counseling for patients, and information provision to other healthcare professionals.

## Figures and Tables

**Table 1:** Social Interactionism Comparisons for Intravenous (IV) and Oral Anticancer Therapies.

	Patients	Oncologists and other Healthcare Professionals
Communication		
IV Therapies	Interactive, face-to-face communication of relevant information	Opportunity to educate and counsel patients directly during therapy; interdisciplinary teamwork Information for cancer care management potentially limited within system
Oral Therapies	Receive extensive amounts of information (e.g., administration, side effects) from oncology team in order to independently deliver, monitor, and safely handle oral anticancer medications. Increased communication with outside providers and practitioners who might not be cancer care specialists	Opportunity to educate and counsel patients at their respective health and digital literacy levels with the intention of better self-management Non-verbal communication of patients unobservable during drug administration with the medication administered at home. Visual ques such as body fatigue, stress, and overall gestalt require patient to initiate conversation.
Care		
IV Therapies	Role in care delivery centered around keeping clinic appointments and reporting events that occur between episodes of care. Outcomes tied to performance of interdisciplinary healthcare team, cancer response to IV therapies, and patient transportation to clinic	Direct observation of intermittent, IV drug administration, checking of patient parameters (e.g., weight, laboratory values), and direct team monitoring and support services
Oral Therapies	Expanded roles for patients and/or informal caregivers (e.g., self-administration of medication, monitoring, contacting healthcare professionals if problems) Increased care provision outside cancer center or oncology practice, e.g., specialty pharmacy	Service delivery diffused across providers within and outside oncology healthcare teams Pharmacists might lack access to patient data needed to check important clinical parameters.
Control		
IV Therapies	Limited control over care delivery	Controlled environment for service and drug delivery More standardized protocols and procedures, safety checks
Oral Therapies	Patients empowered over drug administration, side effect and symptom management	More limited knowledge of medication adherence by patients Potentially reduced opportunities for patient monitoring
Context		
IV Therapies	Care coordination in hospital or clinic Fewer patient responsibilities in direct therapy	Financial and operational mainstay of infusion-based oncologist practice
Oral Therapies	Potentially higher out-of-pocket costs for patients Need to partner with oncologists or pharmacists for timely drug procurement	Less social interactionism among healthcare practitioners Pharmacy benefit management controls
